# Association of serum 25-hydroxyvitamin D and homocysteine “double-risk” status with executive dysfunction in older adults with hypertension

**DOI:** 10.3389/fnut.2025.1718923

**Published:** 2026-01-16

**Authors:** Lili Tan, Linya Zhao, Hongyan Li, Yamin Zhao, Yinyin Chen

**Affiliations:** 1 Department of Geriatrics, Affiliated Hospital of Hebei University, Baoding, Hebei, China; 2 Department of Endocrinology, Affiliated Hospital of Hebei University, Baoding, Hebei, China

**Keywords:** executive function, homocysteine, hypertension, restricted cubic splines, vitamin D

## Abstract

**Background:**

Executive dysfunction is common in adults with hypertension and undermines self-management. Low 25-hydroxyvitamin D [25(OH)D] and elevated homocysteine may damage the endothelium and white matter, while their non-linear dose–response and joint effects on executive dysfunction are unclear.

**Methods:**

We conducted a single-center, prospective, cross-sectional study of inpatients aged ≥60 years with hypertension (November 2023–July 2024). Exposures were vitamin D deficiency [25(OH)D < 20 ng/mL] and hyperhomocysteinemia (hymocysteine ≥15 μmol/L), combined into a four-level dual-risk variable. Executive function was a composite of four tests. Non-linear associations were estimated with logistic regression using restricted cubic splines. Multiplicative interaction was assessed after adjustment for demographic, clinical, renal, lifestyle, supplementation, and seasonal covariates.

**Results:**

Out of 498 individuals screened, 452 participants were analyzed. Vitamin D deficiency was observed in 49% participants, with hyperhomocysteinemia in 34%, dual-risk in 22%, and executive dysfunction in 38% participants. Non-linear associations were evident for both biomarkers (*p*_non-linear ≤0.04). Adjusted odds of executive dysfunction were 1.61 (95% CI 1.2–2.2) at 25(OH)D = 15 vs. 30 ng/mL and 1.4 (1.1–2.0) at homocysteine 18 vs. 10 μmol/L. Relative to neither risk, dual-risk had OR 2.1 (1.5–2.9) with multiplicative interaction (*p* = 0.03) and additive synergy (excess risk due to interaction 0.45, 95% CI 0.06–0.95).

**Conclusion:**

In older hypertensive inpatients, lower 25(OH)D and higher homocysteine are non-linearly associated with executive dysfunction, and co-occurrence confers excess risk beyond individual effects. Assessing both biomarkers may enhance risk stratification and guide trials of combined correction.

## Introduction

Executive dysfunction in older adults with hypertension is a significant public health concern due to its impact on self-management and adverse health outcomes. Hypertension, prevalent in two-thirds of individuals over 60, is a major risk factor for cognitive decline, including executive dysfunction (1, 2). The cognitive decline associated with hypertension often affects the frontal lobe, leading to impairments in decision-making, planning, and organizing (3–5). Furthermore, cognitive impairments can hinder the ability of older adults to engage in health-promoting behaviors (6). Effective management of hypertension in older adults requires an individualized approach that considers cognitive status (1, 7). Therefore, addressing executive dysfunction through interventions such as cognitive training, the use of organizational aids, and patient-centered care strategies could enhance self-management capabilities and improve health outcomes in older adults with hypertension (5, 8).

Low levels of 25-hydroxyvitamin D [25(OH)D] and elevated homocysteine (Hcy) are implicated in mechanisms that contribute to endothelial injury and white matter compromise, which are critical pathways affecting executive control. Elevated homocysteine levels, known as hyperhomocysteinemia (HHcy), are associated with oxidative stress, neuroinflammation, and endothelial dysfunction, all of which contribute to the disruption of the blood–brain barrier and neurodegeneration, thereby increasing the risk of neurovascular diseases such as stroke and vascular dementia (9, 10). HHcy is also linked to the pathogenesis of Alzheimer’s disease through mechanisms such as tau phosphorylation, beta-amyloid aggregation, and neurofibrillary tangle formation, which are exacerbated by oxidative stress and neuroinflammation (3, 10). These processes highlight the role of HHcy in promoting vascular dysfunction and neurodegenerative changes, which are critical to the development of cognitive impairments (11, 12). On the other hand, vitamin D deficiency is associated with increased risks of cardiovascular and neurodegenerative diseases due to its role in maintaining endothelial function and reducing oxidative stress and inflammation (13). Low 25(OH)D levels are correlated with more severe white matter hyperintensities and cognitive decline (14). The neurovascular unit, which includes oligodendrocytes responsible for myelinating axons, is particularly vulnerable to ischemic damage, and vitamin D plays a protective role in maintaining myelin integrity and reducing white matter injury (15). Thus, both low 25(OH)D and elevated Hcy contribute to vascular and neuroinflammatory pathways that compromise myelin integrity and cognitive function (16), and consistent with a cerebrovascular pathway, lower 25(OH)D has also been associated with higher stroke risk (17).

Building on evidence that vitamin D deficiency and hyperhomocysteinemia may affect vascular and white-matter pathways relevant to executive control, our objective was to characterize the associations of serum 25(OH)D and plasma Hcy with executive dysfunction and to test their joint effects on both multiplicative and additive scales in older hypertensive inpatients.

## Methods

### Study design and participants

We enrolled consecutive inpatients aged ≥60 years with physician-diagnosed hypertension between November 2023 and July 2024. The protocol was approved by the institutional ethics committee of the Affiliated Hospital of Hebei University, and all participants provided written informed consent. The study conformed to the Declaration of Helsinki and local regulations for human research.

Patients were ineligible if they had (i) acute stroke or TIA under evaluation or confirmed within the prior 30 days (neuroimaging/neurology note), (ii) current delirium by a standardized screen at the time of cognitive testing (3D-CAM on wards or CAM-ICU in monitored settings), (iii) deep/ongoing sedation [continuous sedative infusion or Richmond Agitation-Sedation Scale (RASS) ≤ −2], (iv) severe sensory/motor barriers precluding valid testing, or (v) inability to communicate in Mandarin/local dialect. Patients with remote ischemic stroke/TIA remained eligible with a history recorded for analysis. Those with transient delirium or recent procedural sedation were deferred and re-approached once delirium screens were negative and washout criteria were met.

### Setting and procedures

Participants were recruited from inpatient medical wards where admissions reflect the evaluation and management of hypertension and common comorbidities. Because acute delirium, active cardiorespiratory decompensation, or sedative medications can transiently affect cognitive performance, we applied prespecified acute-illness safeguards to ensure testing occurred under clinically stable conditions. All testing occurred within 72 h of admission and after acute-illness safeguards: examiners performed a brief neuro check and a delirium screen (3D-CAM/CAM-ICU) immediately pre-test. If an individual was positive, the test was postponed ≥24 h until two consecutive negative screens. To minimize sedative carryover, assessments were scheduled ≥6 h after the last IV sedative/benzodiazepine/opioid dose and ≥12 h after the discontinuation of continuous infusions. Pre-test RASS had to be ≥−1. We abstracted sedation exposure from the medication administration record (any benzodiazepine/opioid in prior 24 h; propofol/dexmedetomidine infusion hours in prior 24 h). We captured acute-infection markers within 24 h of testing—temperature, WBC count, C-reactive protein (CRP), procalcitonin, antibiotic initiation and positive cultures—and cardiorespiratory decompensation proxies (SpO_2_, supplemental O_2_, systolic BP).

### Exposures and laboratory measurements

Primary exposures were serum 25(OH)D and plasma total homocysteine, analyzed in the hospital’s central laboratory with internal quality control during each shift and quarterly external proficiency testing. Serum 25(OH)D was quantified using a chemiluminescent immunoassay on an automated analyzer (Beckman Coulter Access). Plasma total homocysteine was quantified using an enzymatic cycling assay (Diazyme Homocysteine Enzymatic Assay). Serum folate and vitamin B12 were measured using an Abbott ARCHITECT i. Serum creatinine was measured using Creatinine plus (CREP2) on cobas c/COBAS INTEGRA, and eGFR was calculated from creatinine using the Chronic Kidney Disease Epidemiology Collaboration (CKD-EPI) equation. Whole blood was centrifuged for 2 h. Serum/plasma aliquots were analyzed on the day of draw. *A priori*, clinical cut points defined vitamin D deficiency as 25(OH)D < 20 ng/mL (18) and HHcy as homocysteine ≥15 μmol/L. These were combined into a four-level “dual-risk” variable (neither, vitamin D deficiency only, HHcy only, both). To adjust for seasonal variation in 25(OH)D, the calendar month of blood draw was categorized into winter, spring, summer, or autumn for covariate adjustment and sensitivity analyses.

Executive function (EF) was assessed with the Trail Making Test-B (TMT-B), Stroop Color-Word interference, Digit Symbol Substitution (DSST), and Category Fluency. Each raw score was converted to age−/education-adjusted normative *T*-scores (mean 50, SD 10) using published test norms appropriate for our setting. Directionality was aligned so higher *T* indicates better performance. The EF composite *T*-score was the mean of available component *T*-scores when ≥3 tests were valid. The primary binary outcome (executive dysfunction_norm) was composite *T* ≤ 40 (≥1 SD below norms, consistent with mild executive impairment), with *T* ≤ 35 examined as a more stringent threshold in sensitivity analyses. To facilitate comparability with prior inpatient studies, we also constructed a sample-standardized composite *z*-score and defined executive dysfunction_*z* as *z* ≤ −0.3; this moved to a prespecified sensitivity analysis. All scoring was reviewed by a supervising neuropsychologist.

### Covariates

The prespecified adjustment set retained age, sex, education, BMI, systolic BP, diabetes, eGFR modeled continuously with restricted cubic splines, smoking, vitamin D supplementation, B-vitamin supplementation, and season of draw. To address acute-care context, we specified context-robust models that additionally included: history of ischemic stroke/TIA (yes/no), sedation exposure (any benzodiazepine/opioid in prior 24 h or any sedative infusion in prior 24 h; yes/no), and infection burden captured as a composite indicator (fever ≥38.0 °C, WBC ≥ 12 × 10^9^/L, CRP ≥ 10 mg/L, procalcitonin ≥0.25 ng/mL, or new systemic antibiotics within 24 h; yes/no). These variables were abstracted from the EHR around the cognitive testing timestamp.

### Statistical analysis

Baseline characteristics were summarized using means with standard deviations (SDs) or medians with interquartile ranges (IQRs) for continuous variables and counts with whole-number percentages for categorical variables. Group differences were determined using ANOVA or Kruskal–Wallis tests for continuous variables and *χ*^2^ tests for categorical variables, with Benjamini–Hochberg false discovery rate (FDR). Primary analyses modeled associations of 25(OH)D and Hcy with executive dysfunction using multivariable logistic regression with restricted cubic splines (RCS; four knots at Harrell percentiles: 5th, 35th, 65th, and 95th), fixing reference values at 30 ng/mL for 25(OH)D and 10 μmol/L for Hcy. Joint effects were estimated with the four-level exposure, and multiplicative interaction was tested via a product term in fully adjusted models. Additive interaction was quantified from robust Poisson regression with log link and sandwich variance to estimate relative risks and derive the relative excess risk due to interaction (RERI), attributable proportion (AP), and synergy index (S) with 95% CIs via the delta method. Model diagnostics included inspection of leverage and influence (Δ*β* and Cook’s distance), assessment of multicollinearity (variance inflation factors <2), and checks for spline overfitting by comparing Akaike information criteria across knot specifications. Two-sided *α* = 0.05 defined statistical significance, and analyses were performed in R (Version 4.4).

#### Sensitivity analyses

Robustness was evaluated by varying the executive dysfunction threshold to composite *z* ≤ −0.5 and ≤ −0.7, altering spline flexibility by moving knots to quintiles and, separately, refitting with three and five knots, trimming extreme biomarker values by excluding the <1st and >99th percentiles, excluding users of vitamin D and, separately, B-vitamin supplements, and expanding the adjustment set to include folate and vitamin B12 concentrations. The direction and magnitude of primary inferences were stable across these checks.

## Results

### Participant flow and sample characteristics

**Figure 1:**
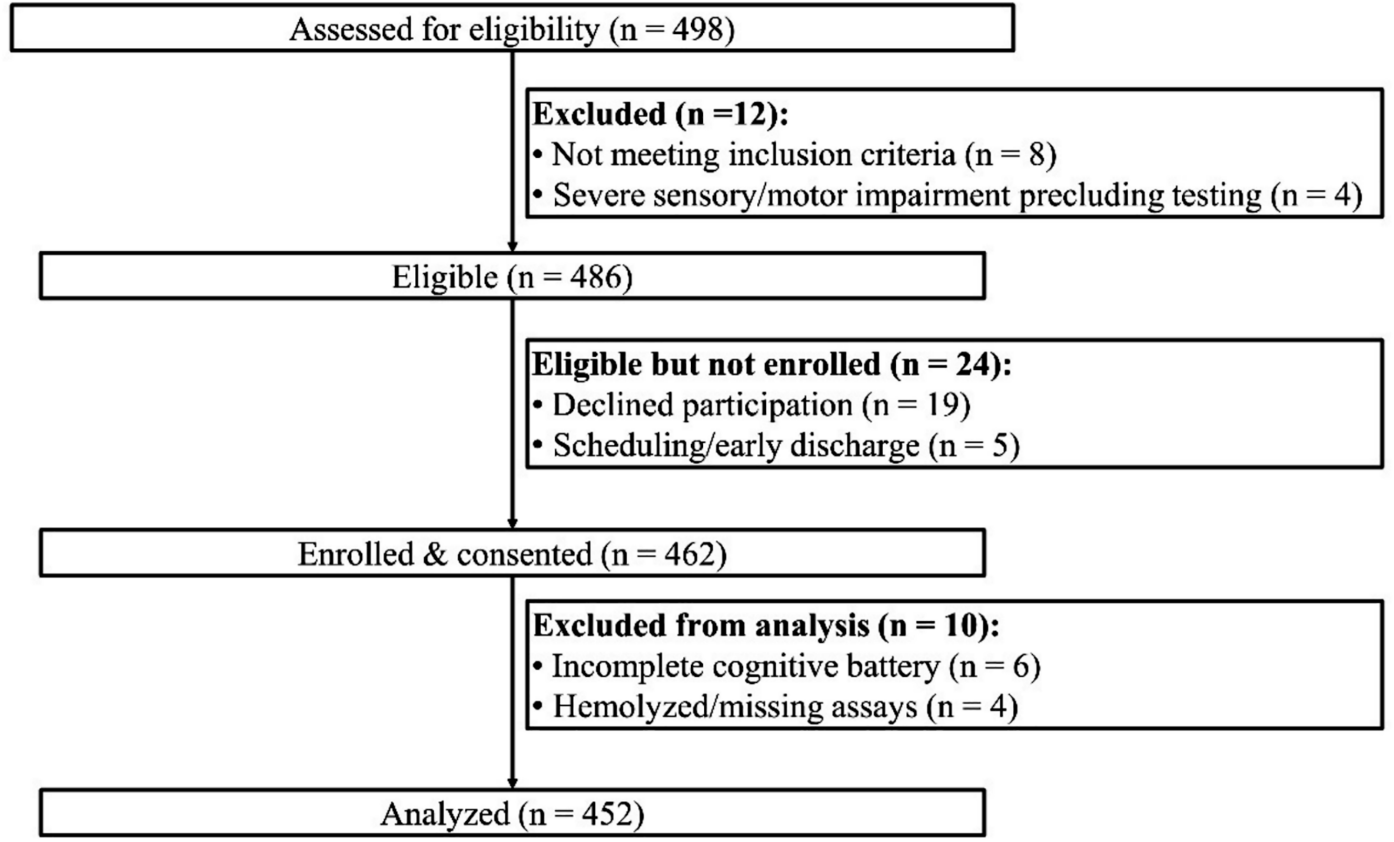
Study flow diagram. Flow of participants from screening to analysis. Final analytic sample *n* = 452.

Of 498 admissions screened, 486 individuals were eligible, 462 consented, and 452 were included in the analytic cohort after exclusion for incomplete cognitive testing (*n* = 6) and unusable assays (*n* = 4) (Figure 1). The mean age was 72 ± 7 years, and 53% were women. Vitamin D deficiency occurred in 49% (*n* = 222), hyperhomocysteinemia in 34% (*n* = 155), and dual-risk in 22% (*n* = 99), while executive dysfunction prevalence was 38% (*n* = 172) (Table 1). Counts across the four dual-risk categories were: neither in 174, vitamin D deficiency in 123, hyperhomocysteinemia in 56, and both in 99 (Table 1).

**Table 1:** Baseline characteristics by vitamin D/homocysteine dual-risk categories.

Characteristic	Overall (*n* = 452)	Neither (*n* = 174)	VitD-only (*n* = 123)	HHcy-only (*n* = 56)	Both (*n* = 99)	*p*	*q*
Age, y (mean ± SD)	72 ± 7	71 ± 6	72 ± 6	74 ± 7	73 ± 7	0.04	0.06
Female, *n* (%)	240 (53%)	100 (57%)	73 (59%)	20 (36%)	47 (48%)	0.003	0.01
Education ≤9 y, *n* (%)	238 (53%)	80 (46%)	70 (57%)	31 (55%)	57 (58%)	0.02	0.04
BMI, kg/m^2^ (mean ± SD)	25 ± 4	25 ± 4	25 ± 4	25 ± 4	24 ± 4	0.11	0.15
SBP, mmHg (mean ± SD)	142 ± 17	141 ± 16	143 ± 17	144 ± 18	145 ± 18	0.09	0.13
Diabetes, *n* (%)	124 (27%)	40 (23%)	36 (29%)	17 (30%)	31 (31%)	0.14	0.18
Chronic kidney disease, *n* (%)	91 (20%)	27 (16%)	23 (19%)	15 (27%)	26 (26%)	0.01	0.03
Current smoker, *n* (%)	98 (22%)	31 (18%)	22 (18%)	20 (36%)	25 (25%)	0.004	0.01
Vitamin B12, pmol/L [median (IQR)]	340 [260–460]	352 [272–466]	338 [252–450]	320 [238–430]	312 [232–420]	0.08	0.12
Folate, ng/mL [median (IQR)]	7.8 [5.6–10.2]	8.0 [6.0–10.5]	7.6 [5.4–9.9]	7.2 [5.1–9.5]	7.0 [4.9–9.3]	0.048	0.07
eGFR, mL/min/1.73 m^2^ [median (IQR)]	74 [61–86]	77 [64–89]	73 [60–85]	70 [57–82]	69 [56–81]	0.007	0.02
25(OH)D, ng/mL [median (IQR)]	21.4 [14.8–28.3]	27.6 [23.1–33.9]	14.6 [11.9–17.6]	26.0 [20.8–31.8]	13.8 [11.1–17.1]	<0.001	<0.001
Homocysteine, μmol/L [median (IQR)]	14.2 [11.0–18.7]	11.2 [9.4–12.7]	12.1 [10.3–14.3]	18.1 [16.2–21.8]	19.3 [16.8–23.7]	<0.001	<0.001
Vitamin D supplement use, *n* (%)	66 (15%)	32 (18%)	8 (7%)	10 (18%)	16 (16%)	0.003	0.01
B-vitamin supplement use, *n* (%)	57 (13%)	19 (11%)	12 (10%)	12 (21%)	14 (14%)	0.03	0.048
Executive dysfunction, *n* (%)	172 (38%)	47 (27%)	48 (39%)	20 (36%)	57 (58%)	<0.001	<0.001

VitD-only, vitamin D deficiency only; HHcy-only, hyperhomocysteinemia only; eGFR, estimated glomerular filtration rate (mL/min/1.73 m^2^).

### Baseline characteristics across dual-risk categories

Participants with both risks had higher current smoking (25%) and CKD (eGFR <60 mL/min/1.73 m^2^) prevalence (26%) and lower eGFR [median 69 mL/min/1.73 m^2^ (56–81)] than those with neither risk [current smoking 18%; CKD 16%; eGFR 77 mL/min/1.73 m^2^ (64–89); *p* = 0.004, 0.01, and 0.007; FDR-adjusted *q* = 0.01, 0.03, and 0.02]. Women were less prevalent in the hyperhomocysteinemia-only group (36%), contributing to overall sex differences (*p* = 0.003; *q* = 0.01). Folate and vitamin B12 trended lower with increasing risk burden (folate *p* = 0.048; *q* = 0.070; B12 *p* = 0.08; *q* = 0.12), and supplementation patterns were imbalanced, with fewer vitamin D supplement users in the vitamin D-deficient only group (7%) and more B-vitamin use in the hyperhomocysteinemia-only group (21%; *p* = 0.003 and 0.03; *q* = 0.01 and 0.048). As constructed, 25(OH)D and Hcy levels showed marked gradients across categories (both *p* < 0.001; *q* < 0.001). Executive dysfunction prevalence rose stepwise from 27% in the neither group to 39% in vitamin D deficiency only, 36% in hyperhomocysteinemia only, and 58% in the dual-risk group (*p* < 0.001; *q* < 0.001), matching *a priori* risk expectations (Table 1).

### Non-linear dose–response associations of biomarkers with executive dysfunction

**Figure 2:**
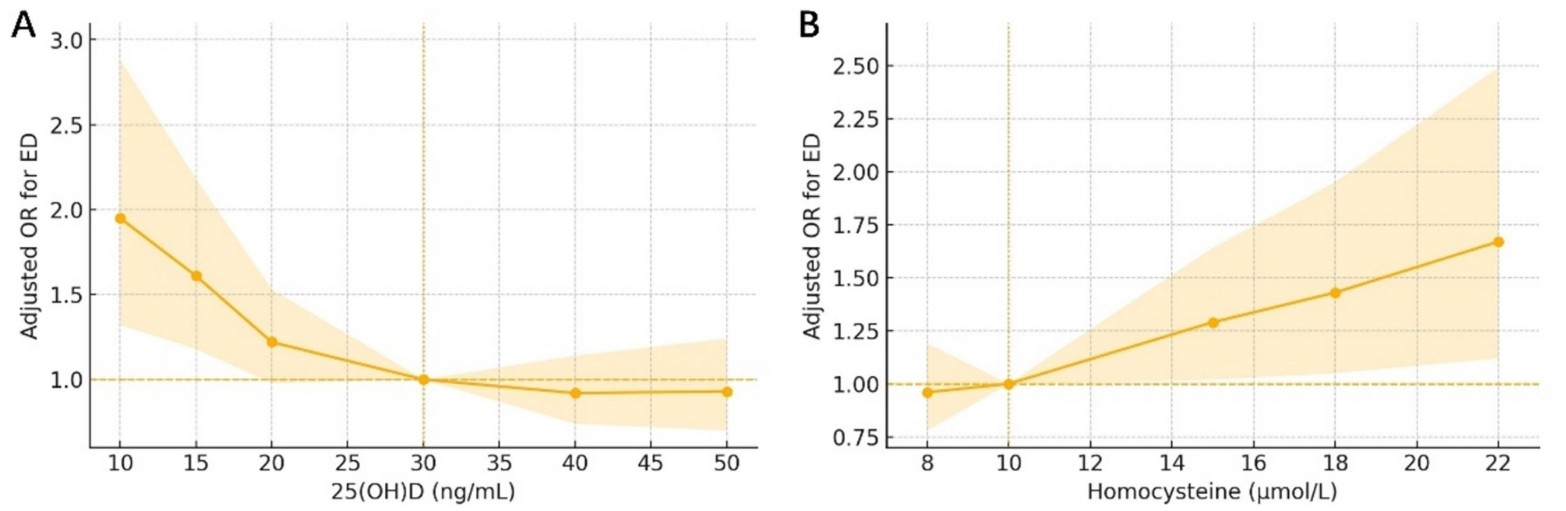
**(A)** Non-linear association of 25(OH)D with executive dysfunction. Adjusted odds ratios (ORs) from logistic models with restricted cubic splines (four knots); reference fixed at 30 ng/mL. Shaded band: 95% CI. Test of non-linearity *p* = 0.02; prespecified contrast 15 vs. 30 ng/mL OR 1.6 (95% CI 1.2–2.2). Covariates as in Methods. **(B)** Non-linear association of homocysteine with executive dysfunction. Adjusted ORs from spline models; reference 10 μmol/L. Shaded band: 95% CI. Test of non-linearity *p* = 0.04; prespecified contrast 18 vs. 10 μmol/L OR 1.4 (95% CI 1.1–2.0). 25(OH)D, 25-hydroxyvitamin D; OR, odds ratio; CI, confidence interval.

Restricted cubic spline models demonstrated non-linear relationships between both biomarkers and executive dysfunction after multivariable adjustment (Table 2, Figure 2). For 25(OH)D, the global non-linearity test was significant (*p* = 0.02; *q* = 0.03), with risk increasing steeply below approximately 20 ng/mL and flattening above 30–40 ng/mL. Relative to 30 ng/mL, the adjusted OR at 15 ng/mL was 1.6 (95% CI 1.2–2.2; *p* = 0.003; *q* = 0.009), and at 10 ng/mL the OR reached 2.0 (1.3–2.9). For homocysteine, non-linearity was also significant (*p* = 0.04; *q* = 0.049), with relatively flat risk at lower concentrations and a monotonic rise beyond approximately 15 μmol/L; the adjusted OR at 18 μmol/L vs. 10 μmol/L was 1.4 (1.1–2.0; *p* = 0.02; *q* = 0.048), increasing to 1.7 (1.1–2.5) at 22 μmol/L.

**Table 2:** Associations of 25(OH)D and homocysteine with executive dysfunction.

Exposure and specification	Adjusted OR (95% CI)	*p*	*q*
Non-linear dose–response (RCS; 4 knots)	—	0.02	0.03
25(OH)D—test of non-linearity	—	0.04	0.049
Hcy—test of non-linearity			
Prespecified contrasts (continuous)	1.6 (1.2–2.2)	0.003	0.009
25(OH)D 15 vs. 30 ng/mL	1.4 (1.1–2.0)	0.02	0.048
Hcy 18 vs. 10 μmol/L			
Binary categories	1.5 (1.1–2.2)	0.02	0.03
Vitamin D deficiency (<20 vs. ≥20 ng/mL)	1.4 (1.0–2.0)	0.045	0.06
Hyperhomocysteinemia (≥15 vs. <15 μmol/L)			
Four-level dual-risk (ref = Neither)	1.4 (1.0–2.0)	0.07	0.08
VitD-only	1.3 (0.8–2.1)	0.23	0.23
HHcy-only	2.1 (1.5–2.9)	<0.001	<0.001
Both (double-risk)	—	0.03	0.04
Multiplicative interaction (product term)	—	0.02	0.03

CI, confidence interval; HHcy, hyperhomocysteinemia; OR, odds ratio; RCS, restricted cubic splines.

### Joint effects of vitamin D deficiency and hyperhomocysteinemia

**Figure 3:**
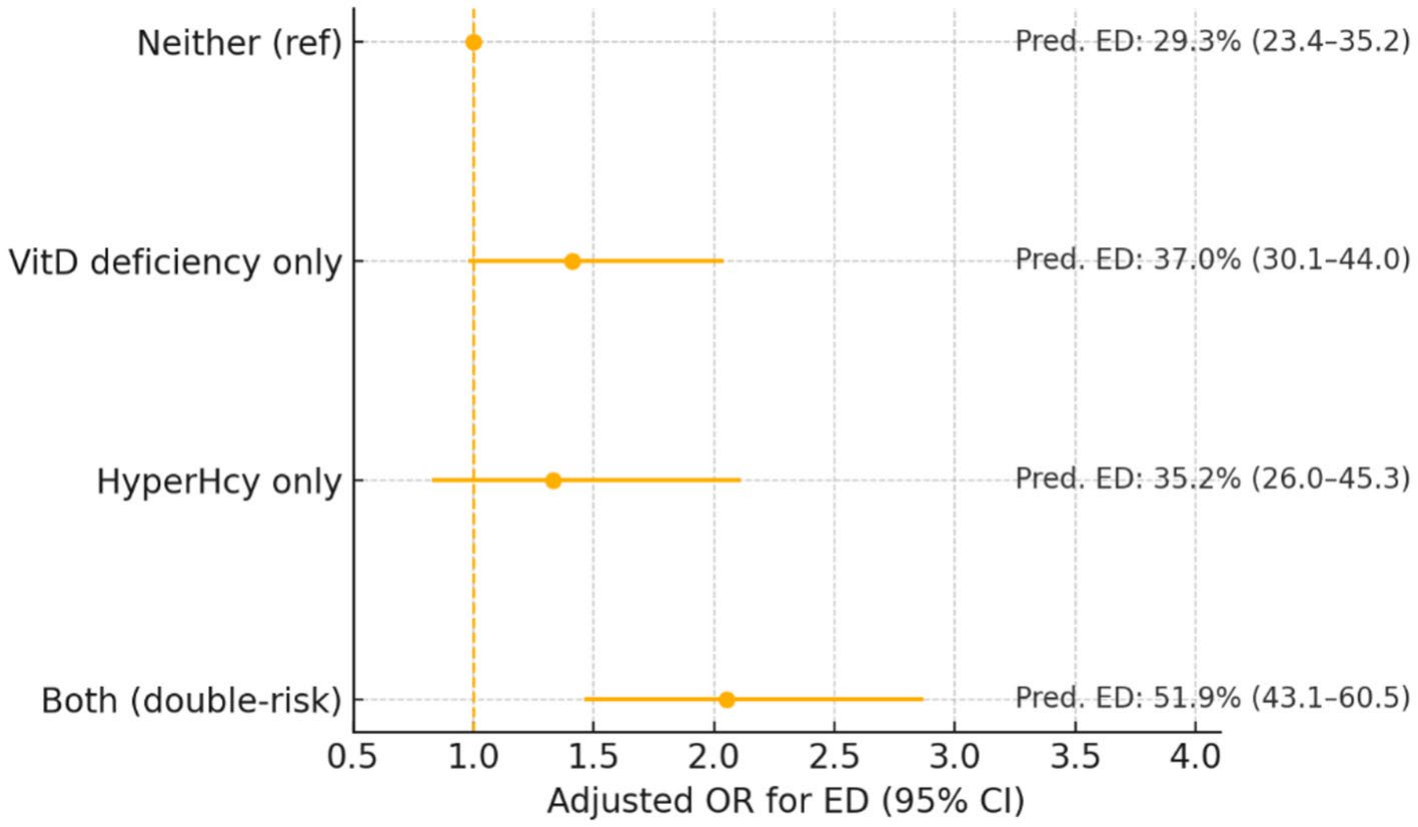
Joint effects of vitamin D deficiency and hyperhomocysteinemia on executive dysfunction. Forest plot shows adjusted ORs (95% CI) for four exposure categories (reference: neither risk), with marginal predicted executive dysfunction probabilities annotated. Double-risk OR 2.1 (95% CI 1.5–2.9); multiplicative interaction *p* = 0.03. executive dysfunction; OR, odds ratio; CI, confidence interval.

Relative to participants with neither risk, those with both risks exhibited more than 2-fold higher odds of executive dysfunction (adjusted OR 2.1, 95% CI 1.5–2.9; *p* < 0.001; *q* < 0.001), whereas point estimates were smaller and not statistically significant for the single-risk categories (vitamin D deficiency only OR 1.4, 95% CI 1.0–2.0; *p* = 0.07; *q* = 0.08; hyperhomocysteinemia only OR 1.3, 95% CI 0.8–2.1; *p* = 0.23; *q* = 0.23). A formal product-term test supported multiplicative interaction between deficiency and hyperhomocysteinemia (*p* = 0.03; *q* = 0.04). Translating relative to absolute risk at the sample-mean covariate profile, predicted executive dysfunction probabilities were 29% (95% CI 23–35) for neither, 37% (30–44) for vitamin D deficiency only, 35% (26–45) for hyperhomocysteinemia only, and 52% (43–61) for both (Figure 3).

### Additive interaction

On the additive scale, robust Poisson models indicated positive biological interaction between vitamin D deficiency and HHcy (Table 3). The relative excess risk due to interaction (RERI) was 0.45 (95% CI 0.06–0.95; *p* = 0.03; *q* = 0.04), the attributable proportion (AP) was 0.22 (0.03–0.39; *p* = 0.03; *q* = 0.04), and the synergy index (S) was 1.5 (1.1–2.1; *p* = 0.03; *q* = 0.04), indicating that approximately one-fifth of risk among dually exposed patients reflects excess risk beyond the sum of individual effects (Table 3, Figure 3).

**Table 3:** Additive interaction between vitamin D deficiency and hyperhomocysteinemia for executive dysfunction.

Metric (ref = Neither)	Estimate (95% CI)	*p*	*q*
RERI (excess risk due to interaction)	0.45 (0.06–0.95)	0.03	0.04
Attributable proportion (AP)	0.22 (0.03–0.39)	0.03	0.04
Synergy index (S)	1.5 (1.1–2.1)	0.03	0.04

Executive dysfunction; CI, confidence interval; RERI, relative excess risk due to interaction; AP, attributable proportion; S, synergy index.

### Sensitivity analyses

Robustness checks confirmed that the main inferences did not depend on the outcome definition, kidney-function covariate, or inpatient context (Supplementary Tables S1, S2) (Supplementary Tables S1, S2). Using stricter executive dysfunction thresholds (*z* ≤ −0.5 and *z* ≤ −0.7) yielded the same non-linear patterns for both biomarkers [25(OH)D non-linearity *p* = 0.02–0.01; Hcy *p* = 0.04–0.03; all *q* ≤ 0.05] with contrasts similar to the primary analysis—25(OH)D 15 vs. 30 ng/mL OR 1.6–1.7 and Hcy 18 vs. 10 μmol/L OR 1.4–1.5—and preserved the joint “double-risk” association (OR 2.1–2.2) together with multiplicative interaction (*p* = 0.03–0.02; *q* ≤ 0.05) and positive additive interaction (RERI 0.47–0.55) (Supplementary Tables S1, Panel A). Representing renal function as CKD (eGFR<60 mL/min/1.73 m^2^) instead of the eGFR-spline produced materially similar estimates [25(OH)D OR 1.6; Hcy OR 1.4; double-risk OR 2.0; product-term *p* = 0.04; RERI 0.44], supporting the choice to model kidney function once, continuously (Supplementary Tables S1, Panel B). Context-robust models that added prior stroke/TIA, recent sedation exposure, and infection burden, as well as a restricted analysis excluding any sedation/infection in the prior 24 h, closely matched the primary results [25(OH)D OR 1.6–1.7; Hcy OR 1.4–1.5; double-risk OR 2.0–2.1; product-term *p* = 0.03–0.02; RERI 0.43–0.47], indicating that acute-care factors did not account for the observed non-linear associations or the excess risk in the double-risk group (Supplementary Table S2).

## Discussion

In this cross-sectional cohort study, we found that both lower 25(OH)D and higher Hcy were related to executive dysfunction in distinctly non-linear patterns. Crucially, when both abnormalities co-occurred (“double-risk”), the association exceeded individual effects: compared with patients with neither risk, the double-risk group had more than 2-fold higher odds of executive dysfunction.

Placed alongside prior studies, our results extend observational associations between low 25(OH)D and poorer executive performance and faster decline, and between higher Hcy and heavier cSVD/white-matter burdens, by revealing where along each continuum risk accelerates and by quantifying biological interaction between the two pathways (19–25). Studies in community and patient cohorts link low 25(OH)D to worse cognition and increased dementia risk, while neuroimaging reports connect low 25(OH)D and high Hcy to more white-matter hyperintensities and microstructural compromise—lesions that map onto executive control networks (19–25). Methodologically, modeling with restricted cubic splines avoids the information loss and bias of linear or dichotomized exposures and makes the inflection zones explicit, and estimating additive-scale interaction (RERI, attributable proportion, synergy index) addresses whether the “double-risk” exceeds the sum of individual effects (26–28). Our spline-based contrasts and significant additive/multiplicative interaction, therefore, add granularity beyond much of the existing vitamin D/Hcy–cognition literature.

Clinically, our data support early inpatient assessment of both biomarkers in older adults with hypertension to refine cognitive risk stratification and to identify patients most likely to benefit from intervention (18, 29–33). In hypertensive populations, achieving 25(OH)D status has been linked to blood pressure measures in community supplement programs (34), and meta-analytic evidence suggests vitamin D supplementation may modestly reduce systolic blood pressure in hypertensive individuals with hypovitaminosis D (35). For homocysteine-lowering, the China Stroke Primary Prevention Trial (CSPPT) randomized hypertensive adults to enalapril plus folic acid vs. enalapril alone and reported reduced first stroke, whereas the Heart Outcomes Prevention Evaluation-2 (HOPE-2) trial tested folic acid/B-vitamin therapy in vascular disease and reported mixed cardiovascular outcomes but fewer strokes (36, 37). Recent randomized controlled trial evidence suggests vitamin D supplementation does not improve executive function in older adults with mild cognitive impairment (38), and a recent meta-analysis suggests B-vitamin supplementation may have small effects on global cognitive function in older adults (39). Together, these data motivate factorial trials testing simultaneous vitamin D repletion and homocysteine-lowering on executive outcomes (and MRI white-matter endpoints) on top of guideline blood pressure control, enriched for patients who exhibit the dual-risk pattern.

Our findings point to a vascular-white-matter pathway linking both low 25(OH)D and elevated Hcy to executive dysfunction in older adults with hypertension. Risk rose steeply once 25(OH)D dropped into deficient ranges and as Hcy exceeded clinically used cutpoints, consistent with the biology that vitamin D restrains renin–angiotensin signaling and supports endothelial nitric oxide bioavailability, whereas Hcy impairs the nitric-oxide pathway via asymmetric dimethylarginine, promoting endothelial injury and prothrombotic tone (29–32). In cerebral small-vessel disease, endothelial failure, blood–brain barrier leak, and hypoperfusion render frontal–subcortical white matter especially vulnerable, providing a substrate for executive dysfunction (40–43). This mechanistic arc explains the non-linear dose–response we observed for both biomarkers and the excess risk when deficiency and hyperhomocysteinemia co-occur, with predicted executive dysfunction probabilities rising from 29% (neither risk) to 52% (double-risk) in our cohort. The inflection zones align with widely adopted thresholds [25(OH)D < 20 ng/mL; Hcy ≥ 15 μmol/L] at which endothelial and white-matter injury patterns intensify (18, 29–33, 40–43).

Nonetheless, limitations warrant a cautious interpretation of our findings. The cross-sectional design precludes causal inference and leaves open reverse causation (e.g., illness severity influencing nutritional status or homocysteine). Residual confounding remains possible despite adjustment (e.g., sun exposure, dietary folate/B12, parathyroid hormone, and inflammatory status). Findings derive from a single-center inpatient population and may not generalize to community or outpatient settings. Single-time biomarker measurements are vulnerable to short-term variability and seasonality despite seasonal adjustment and sensitivity analyses. Complete-case primary models, with multiple imputation as a check, reduce but cannot eliminate bias from missingness. Taken together, these strengths support the credibility of the observed non-linear associations and dual-scale interaction, while the limitations emphasize the need for multicenter, longitudinal studies with repeated biomarker assessments and mechanistic imaging to establish temporality and transportability.

In older hypertensive inpatients, lower 25-hydroxyvitamin D and higher homocysteine relate to executive dysfunction in non-linear patterns, and their co-occurrence confers excess risk beyond individual effects. Early inpatient assessment of both biomarkers can refine cognitive risk stratification and highlight patients most likely to benefit from targeted therapy. These findings support multicenter longitudinal studies and randomized factorial trials testing combined vitamin D repletion and homocysteine-lowering alongside guideline blood-pressure control.

## Data Availability

The raw data supporting the conclusions of this article will be made available by the authors, without undue reservation.
