# Supplementary material for: Association of serum 25-hydroxyvitamin D and homocysteine “double-risk” status with executive dysfunction in older adults with hypertension
Source: Front Nutr. 2026 Jan 16;12:1718923. doi: 10.3389/fnut.2025.1718923 (PMC12855134; doi:10.3389/fnut.2025.1718923)
Supplement: Supplementary file 1 [file Table_1.docx]

**Supplementary Table S1. Outcome definition and kidney function representation (minimal robustness set)**

**Panel A. Alternative ED thresholds**

| **Metric** | **Primary ED (z ≤ −0.3)** | **ED (z ≤ −0.5)** | **ED (z ≤ −0.7)** |
| --- | --- | --- | --- |
| **25(OH)D non‑linearity p (q)** | 0.02 (0.03) | 0.02 (0.03) | 0.02 (0.03) |
| **Hcy non‑linearity p (q)** | 0.04 (0.049) | 0.046 (0.049) | 0.04 (0.047) |
| **OR: 25(OH)D 15 vs 30 ng/mL** | 1.6 (1.2–2.2), p=0.003, q=0.009 | 1.6 (1.1–2.2), p=0.008, q=0.02 | 1.7 (1.2–2.5), p=0.005, q=0.01 |
| **OR: Hcy 18 vs 10 µmol/L** | 1.4 (1.1–2.0), p=0.02, q=0.048 | 1.4 (1.0–2.0), p=0.04, q=0.049 | 1.5 (1.0–2.2), p=0.04, q=0.049 |
| **Double‑risk vs Neither (OR)** | 2.1 (1.5–2.9), p<0.001, q<0.001 | 2.1 (1.4–3.1), p<0.001, q=0.002 | 2.2 (1.4–3.5), p<0.001, q=0.002 |
| **Multiplicative interaction p (q)** | 0.03 (0.04) | 0.03 (0.045) | 0.03 (0.04) |
| **RERI** | 0.45 (0.06–0.95), p=0.03, q=0.04 | 0.47 (0.05–1.03), p=0.03, q=0.04 | 0.55 (0.07–1.16), p=0.02, q=0.04 |

**Panel B. Kidney function representation**

| **Kidney covariate** | **25(OH)D 15 vs 30 ng/mL OR (95% CI)** | **Hcy 18 vs 10 µmol/L OR (95% CI)** | **Double‑risk OR (95% CI)** | **Product‑term p (q)** | **RERI (95% CI)** |
| --- | --- | --- | --- | --- | --- |
| **eGFR (RCS, 4 knots)** | 1.6 (1.2–2.2) | 1.4 (1.1–2.0) | 2.1 (1.5–2.9) | 0.03 (0.04) | **0.45 (0.06–0.95)** |
| **CKD** **(eGFR<60** mL/min/1.73 m^2^**)** | 1.6 (1.2–2.2) | 1.4 (1.0–1.9) | 2.0 (1.4–2.8) | 0.04 (0.046) | 0.44 (0.05–0.94) |

Note: The primary definition of executive dysfunction was the age-/education-adjusted composite T-score ≤40; z-score definitions are provided as sensitivity analyses.
