# Supplementary material for: Association of serum 25-hydroxyvitamin D and homocysteine “double-risk” status with executive dysfunction in older adults with hypertension
Source: Front Nutr. 2026 Jan 16;12:1718923. doi: 10.3389/fnut.2025.1718923 (PMC12855134; doi:10.3389/fnut.2025.1718923)
Supplement: Supplementary file 2 [file Table_2.docx]

**Supplementary Table S2. Acute‑care context robustness (stroke/TIA, sedation, infection)-minimal model set**

| **Model** | **25(OH)D 15 vs 30 ng/mL OR (95% CI)** | **Hcy 18 vs 10 µmol/L OR (95% CI)** | **Double‑risk OR (95% CI)** | **Product‑term p (q)** | **RERI**  **(95% CI)** |
| --- | --- | --- | --- | --- | --- |
| **Primary**  **(base covariates)** | 1.6 (1.2–2.2) | 1.4 (1.1–2.0) | 2.1 (1.5–2.9) | 0.03 (0.04) | **0.45 (0.06–0.95)** |
| **+ Stroke/TIA history** | 1.6 (1.2–2.2) | 1.4 (1.0–1.9) | 2.0 (1.5–2.9) | 0.03 (0.04) | 0.45 (0.06–0.95) |
| **+ Sedation & infection** | 1.6 (1.2–2.2) | 1.4 (1.0–1.9) | 2.0 (1.4–2.9) | 0.04 (0.046) | 0.43 (0.04–0.93) |
| **Restricted**  **(no sedation/infection)** | 1.7 (1.2–2.3) | 1.5 (1.1–2.0) | 2.1 (1.5–3.0) | 0.03 (0.04) | 0.47 (0.07–1.02) |
